# The Efficacy of Colistin Combined with Amikacin or Levofloxacin against Pseudomonas aeruginosa Biofilm Infection

**DOI:** 10.1128/spectrum.01468-22

**Published:** 2022-09-14

**Authors:** Yuhang Wang, Chunsun Li, Jin Wang, Nan Bai, Huan Zhang, Yulong Chi, Yun Cai

**Affiliations:** a Center of Medicine Clinical Research, Department of Pharmacy, Medical Supplies Center, PLA General Hospital, Beijing, People’s Republic of China; b Laboratory of Department of Pulmonary and Critical Care Medicine, PLA General Hospital, Beijing, People’s Republic of China; INTHERES

**Keywords:** *Pseudomonas aeruginosa*, biofilm, colistin, levofloxacin, amikacin, meropenem

## Abstract

Pseudomonas aeruginosa (PA) biofilm infection is clinically prevalent and difficult to eradicate. In the present work, we aimed to evaluate the *in vitro* and *in vivo* efficacy of colistin (COL)-based combinations against PA biofilm. MICs and fractional inhibitory concentration indexes (FICIs) of four antibiotics (COL, amikacin, levofloxacin, and meropenem) to bioluminescent strain PAO1, carbapenem-resistant PAO1 (CRPAO1), and clinically isolated strains were assessed. Minimal biofilm eradication concentrations (MBECs) of monotherapy and combinations were examined by counting the live bacteria in biofilm, accompanied by visual confirmation using confocal laser-scanning microscopy. An animal biofilm infection model was established by implanting biofilm subcutaneously, and the therapeutic effect was evaluated according to the change in luminescence through a live animal bio-photonic imaging system. *In vitro*, even combined with 4 or 8 mg/L COL, meropenem needed to reach 128 or 256 mg/L to eradicate the biofilm. Moreover, 2 mg/L COL combined with 32 mg/L amikacin or 4-8 mg/L levofloxacin could kill the PAO1 and CRPAO1 in biofilm within 24 h. *In vivo*, COL combined with amikacin or levofloxacin could shorten the eradication time of biofilm than monotherapy. For PAO1 biofilm, combination therapy could eradicate the biofilm in all mice on the 5th day, whereas monotherapy only eradicated biofilms in almost half of the mice. For CRPAO1 biofilm, the biofilm eradication rate on the 6th day in the COL+ amikacin, amikacin, or COL alone regimen was 90%, 10%, or 40%, respectively. COL combined with levofloxacin did not show a better effect than each individual antibiotic. COL-based combinations containing levofloxacin or amikacin were promising choices for treating PA biofilm infection.

**IMPORTANCE** Infections associated with PA biofilm formation are extremely challenging. When monotherapy fails to achieve optimal efficacy, combination therapy becomes the last option. After evaluating multiple drug combinations through a series of experiments *in vitro* and *in vivo*, we confirmed that colistin-based combinations containing levofloxacin or amikacin were promising choices for treating PA biofilm infection. The efficacy of these combinations derives from the different bactericidal mechanisms and the bacterial susceptibility to each antibiotic. This study provided a new regimen to solve the incurable problem of biofilm by using COL combined with other antibiotics.

## INTRODUCTION

Pseudomonas aeruginosa (PA) biofilm infections commonly exist in cystic fibrosis, burn wounds, and indwelling devices, resulting in high morbidity and mortality (1). The bacteria in biofilm are protected by the highly hydrated extracellular polymeric substances, which can escape host immune attacks and become up to 1,000-fold more resistant to conventional antibiotics (2). Antibiotic tolerance in biofilms may not be hereditary, which can be attributed to the diversity of metabolism between cells in biofilms and the protection of antibiotics by the biofilm matrix. In the case of genetic resistance, frequent horizontal gene transfer in the biofilm will quickly lead to the spread of antimicrobial resistance genes (3). The increasing number of antibiotic- tolerant bacteria in biofilms provides an ever-increasing challenge for the treatment (4).

Colistin (COL) shows an excellent bactericidal potency and spectrum against aerobic Gram-negative bacilli, including most Enterobacterales, Acinetobacter baumannii, and PA (5). In a biofilm, things are complicated. There is a significant spatial and physiological heterogeneity in the structure of biofilms, which may range from a flat homogeneous cell layer to a highly organized biofilm with mushroom-shaped microcolonies separated by water channels (6, 7). COL affects bacteria with lower metabolic activity in the deep layers, while the metabolically active cells in the outer layers may survive (8).

Therefore, the combined use of antibiotics that can kill bacteria in different states may solve the problem. It can also effectively reduce the production of drug-resistant bacteria. In the present study, we evaluated the anti-biofilm ability of COL-based combinations containing three different classes of antibiotics [levofloxacin (LEV), amikacin (AMI), and meropenem (MER)] *in vivo* and *in vitro*.

## RESULTS

### MICs, MBCs, and FICIs on planktonic PA *in vitro*.

Tables 1 and 2 summarize the MICs and FICIs of the tested antibiotics. PAO1 was susceptible to four antibiotics, while carbapenem-resistant PAO1 (CRPAO1) was resistant to MER. C22 was resistant to LEV and AMI. Except that the COL+MER combination showed partial synergy on PAO1 and CRPAO1 with an FICI of 0.75, other combinational effects were indifferent according to the FICIs.

**TABLE 1:** MICs, MBCs, and FICIs of antibiotics against three PA strains^*a*^

	MIC/MBC (mg/L)				FICI		
Strains	COL	LEV	AMI	MER	COL+LEV	COL+AMI	COL+MER
C22	2/4	4/8	>1024/-	2/4	2	-	1
PAO1	1/2	0.5/1	2/8	1/2	2	1	0.75
CRPAO1	2/4	1/8	4/16	16/64	1	1	0.75

a CLSI breakpoints: MER, 2 mg/L; COL,  2 mg/L; AMI, 32 mg/L; LEV, 2 mg/L. -, no data available.

**TABLE 2:** MICs and FICIs of antibiotics against 21 PA strains^*a*^

Strains	MIC/susceptible	COL	LEV	AMI	MER	FICI	COL+LEV	COL+AMI	COL+MER
Clinical isolated(*n* = 21)	MIC_50_ (mg/L) MIC_90_ (mg/L) Susceptible (%)	0.5 4 95.2	0.5 4 81.0	2 2 76.2	4 4 52.4	FICI ≤ 0.5	1	0	4
						0.5 < FICI < 1	2	9	4
						1 ≤ FICI ≤ 2	18	12	13
						2 < FICI	0	0	0

a CLSI breakpoints: MER, 2 mg/L; COL,  2 mg/L; AMI, 32 mg/L; LEV, 2 mg/L.

For 21 clinically isolated strains, COL combined with LEV, AMI, or MER showed indifference to most strains and synergy or partial synergy to a small part of strains. No antagonism was observed.

### Synergistic activity of three antimicrobial combinations to PA biofilm *in vitro*.

Table 3 shows the MBECs of COL, LEV, MER, and AMI to 1-day-grown, 3-day-grown, and 7-day-grown PA biofilms. The MBECs of each antibiotic against 3-day-grown and 7-day-grown biofilms were higher than the 1-day-grown biofilm. After combination, the MBECs of COL were decreased to 1 to 4 mg/L, and the MBECs of LEV or AMI were decreased to half or a quarter of the drug alone. However, the MER concentration in the combinations remained higher than 128 mg/L. Therefore, the COL+MER combination was not evaluated in the subsequent studies.

**TABLE 3:** MBECs of antibiotics against PA biofilm^*a*^

Strains	Biofilm growth age	MBEC (mg/L)				MBEC-combination (mg/L)		
		COL	LEV	AMI	MER	COL+LEV	COL+AMI	COL+MER
C22	1 day	8	16	-	>256	2 + 8	-	4 + 256
	3 day	16	64	-	-	2 + 16	-	-
	7 day	16	64	-	-	2 + 32	-	-
PAO1	1 day	8	4	32	>256	2 + 2	4 + 8	4 + 128
	3 day	64	8	64	-	2 + 4	2 + 32	-
	7 day	64	8	64	-	2 + 4	2 + 32	-
CRPAO1	1 day	16	8	64	>256	1 + 4	1 + 32	8 + 128
	3 day	32	16	64	-	2 + 8	2 + 32	-
	7 day	32	16	64	-	2 + 8	4 + 32	-

a CLSI breakpoints: MER, 2 mg/L; COL,  2 mg/L; AMI, 32 mg/L; LEV, 2 mg/L. -, no data available.

In Fig. S1A to S3A, we marked the concentration of each antibiotic according to the CLSI intermediate or susceptible point to demonstrate whether the concentrations could be achieved *in vivo* using the recommended dosage. For PAO1 biofilm, 2 mg/L COL combined with 2 or 4 mg/L LEV could kill the bacteria in 1-day-grown or 3/7-day-grown biofilm, respectively. Likewise, 32 mg/L AMI alone or in combination with 2 mg/L COL could eradicate the 1-day-grown or 3/7-day-grown biofilm, respectively (Fig. S1A). For 1/3-day-grown CRPAO1 biofilm, the COL+AMI group had a good effect at the concentration of 2 + 32 mg/L, while the MBEC-combination at 4 + 32 mg/L could eradicate the 7-day-grown biofilm. Besides, 2 mg/L COL combined with 4 or 8 mg/L LEV could eradicate the 1-day-grown or 3/7-day-grown biofilm, respectively (Fig. S2A). For the C22 biofilm, since the MIC of AMI > 1,024 mg/L, only the combination of LEV and COL was tested. When COL was 2 mg/L, only LEV ≥ 8 mg/L could kill bacteria in biofilm within 24 h (Fig. S3A). CLSM images showed the same synergistic activity with the MBECs (Fig. S1B-3B).

### Synergistic effects of antimicrobial combinations on PA biofilm *in vivo*.

**FIG 1:**
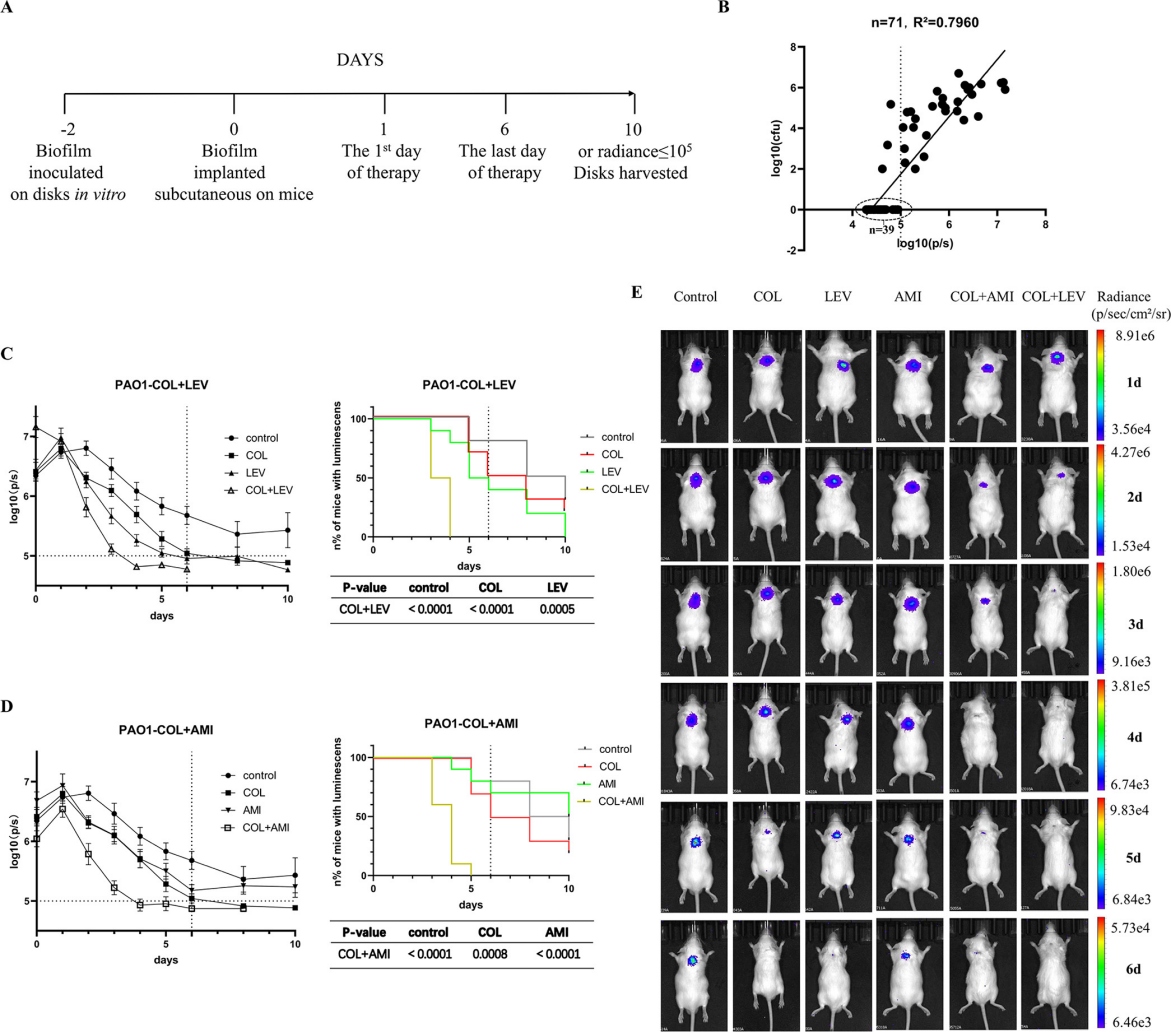
*In vivo* synergistic activity of antimicrobial combinations to PAO1 biofilm. (A) Timeline of biofilm experiments. (B) Correlation between the bacterial counts in biofilm and the radiance (*n* = 71). (C–D) The changes of radiances every day and the curve of the number of mice with detectable luminescence. (E) Dorsal images of representative PAO1-infected mice. COL (COL 20 mg/kg/12 h); LEV (LEV 32 mg/kg/24 h); AMI (AMI 135 mg/kg/24 h); COL+LEV (COL 20 mg/kg/12 h plus LEV 32 mg/kg/24 h); and COL+AMI (COL 20 mg/kg/12 h plus AMI 135 mg/kg/24 h). Results represent means ± SEM. *, *P* < 0.05; **, *P* < 0.01; ***, *P* < 0.001.

**FIG 2:**
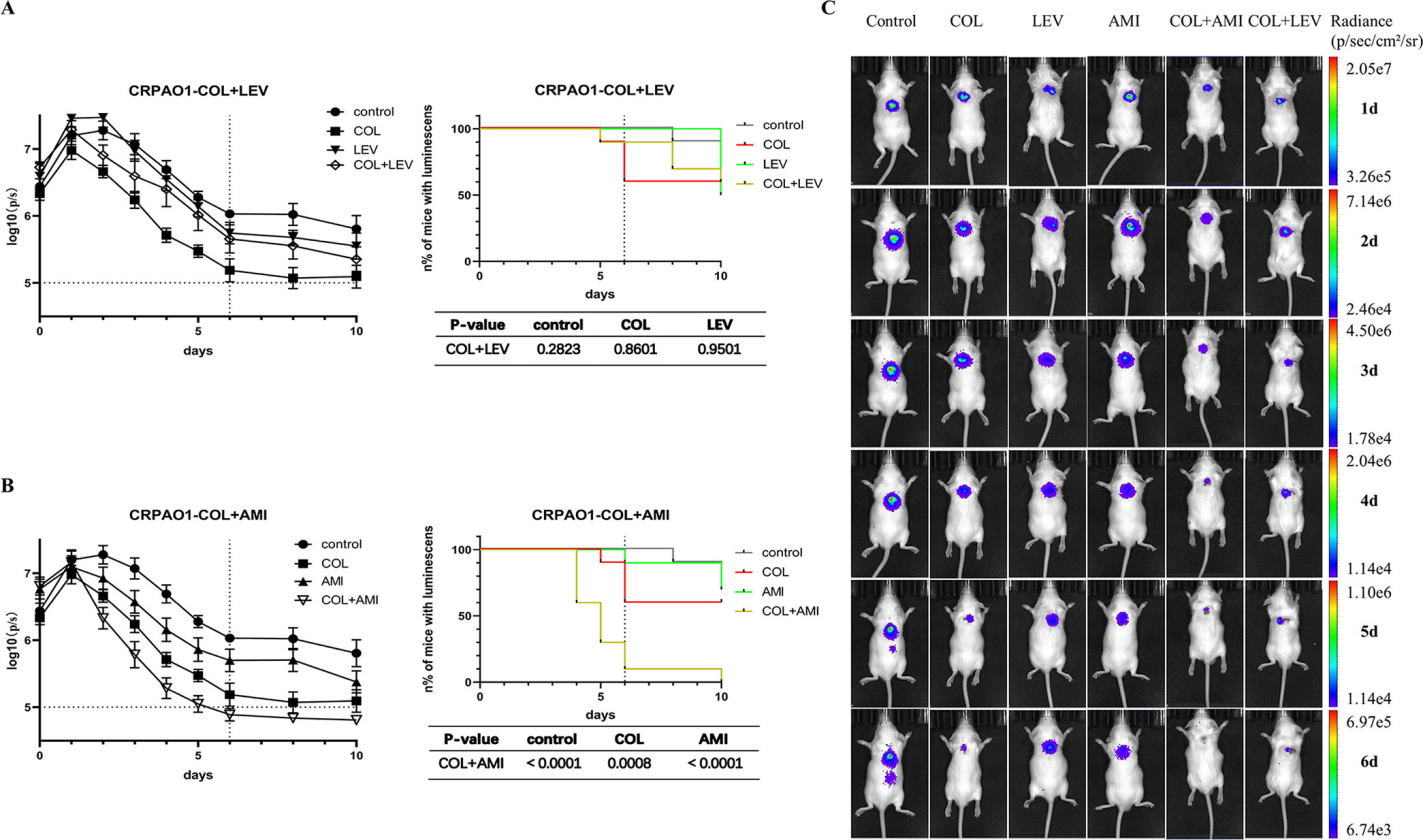
*In vivo* synergistic activity of antimicrobial combination to CRPAO1 biofilm. (A–B) The changes of radiances every day and the curve of the number of mice with detectable luminescence. (C) Dorsal images of representative CRPAO1-infected mice challenged. LEV (LEV 64 mg/kg/24 h); COL (COL 20 mg/kg/12 h); AMI (AMI 135 mg/kg/24 h); COL+LEV (COL 20 mg/kg/12 h plus LEV 64 mg/kg/24 h); and COL+AMI (COL 20 mg/kg/12 h plus AMI 135 mg/kg/24 h). Results represent means ± SEM. *, *P* < 0.05.

The correlation between the bacterial counts in biofilm and the radiance was good (*n* = 71, r^2^ = 0.7960, *P* < 0.00001, Fig. 1B). No living bacteria were found in biofilm when the total flux was less than 10^5^. The CFU counts on the 10th day were listed in Fig. S4. In general, the results of CFU were consistent with the luminescence. Since the implant of some mice was exposed due to a ruptured suture on the 10th day, the CFU counts in those biofilms were omitted. Fig. 1E and Fig. 2C illustrate the dorsal images of representative mice challenged every day.

For PAO1 biofilm-infected mice, luminescence could not be detected in 50% and 60% of mice in the COL and LEV groups on the 6th day, respectively, while such a proportion was only 30% in the AMI group. In addition, the luminescence of all mice in the COL+LEV and COL+AMI groups could not be detected on the 4th day and 5th day, respectively. The photons of the combinations also could decrease faster than antibiotics alone. After the end of therapy, the photons of each group showed a slightly decreasing trend (Fig. 1C and D).

For CRPAO1, no obvious therapeutic effects were observed on the monotherapy regimen (luminescence could be detected in more than 60% of mice on the 6th day). Only the COL+AMI group showed good therapeutic effects on the 6th day, and the luminescence could not be detected in 90% of mice. Besides, the average photons/second was decreased to the lower limit of detection. The COL+LEV group showed no significant difference compared with the control and monotherapy groups (*P* > 0.05). At the end of therapy, the photons of each group continued decreasing a little (Fig. 2A and B).

**FIG 3:**
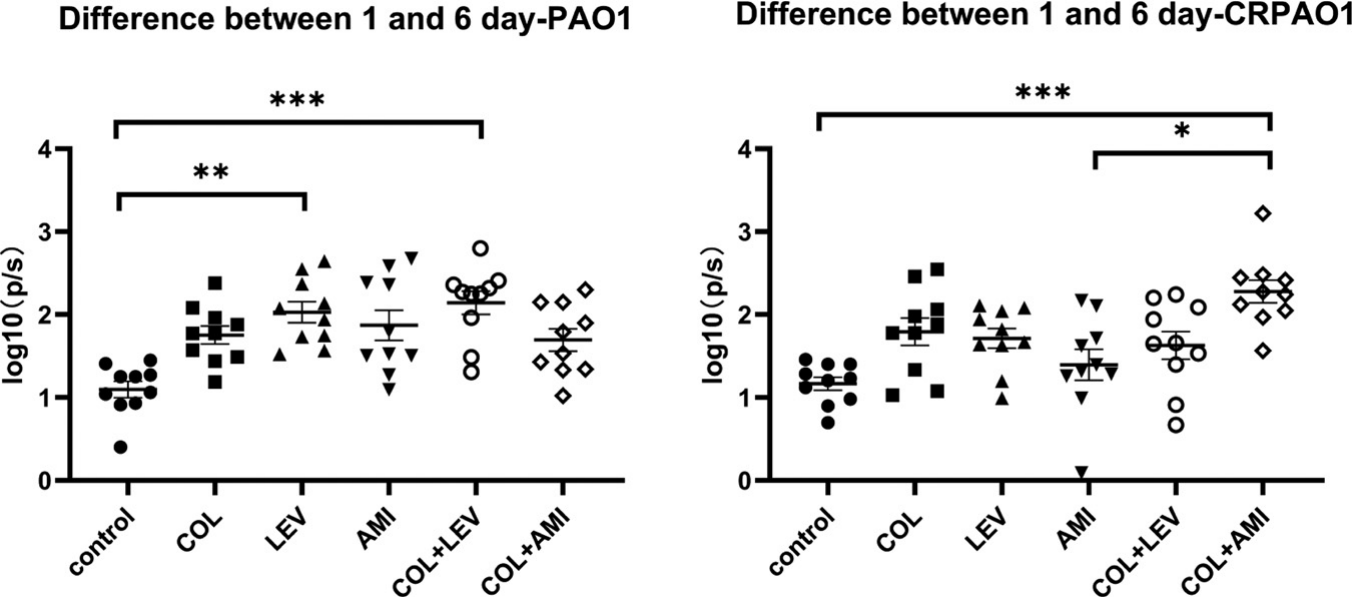
The changes of the photons from the 1st day to the 6th day. Results represent means ± SEM. *, *P* < 0.05; **, *P* < 0.01; ***, *P* < 0.001.

To reduce the influence of the initial bacterial amount in biofilm, we analyzed the changes of the photons between the 1st day and the 6th day (Fig. 3). For PAO1, LEV and COL+LEV could significantly reduce the number of bacteria in the biofilm compared with control group. For CRPAO1, only the COL+AMI group can reduce bacterial numbers in biofilms. Moreover, the COL+AMI combination had better effects than AMI alone.

### Renal histopathological examination.

After 5 days of the administration, no abnormality was found in the control and AMI groups. A small amount of tubular epithelial cell vacuolar degeneration and slight tubular dilation were observed in the COL and COL+AMI groups, while the difference between the two groups was not noticeable.

## DISCUSSION

Nowadays, it is still challenging to treat biofilm infection. Traditional antibiotics require high concentrations to kill bacteria in biofilms *in vitro*, while such a concentration is difficult to achieve *in vivo*. Although the antibiotics we chose showed high sensitivity rates to planktonic PA in our results and surveillance program, the MBECs of single drugs were many times higher than their breakpoints (9, 10). The MBECs of our single drugs to PAO1 immature biofilm were eight times to >256 times of MICs. For mature biofilms (≥3 days), MBECs were even higher. Moreover, it has been found that carbapenem resistance has increased globally, and in most countries, CR-PA represents 10 to 50% of the population (11). It is challenging to administer an appropriate empirical therapy in cases of PA infections resistant to carbapenem (12). In order to treat infections caused by CR-PA, even in the case of extensively drug-resistant (XDR) pathogens, some antibiotics can be combined with COL to exert a synergistic effect or to decrease the possible adverse events of each drug (13). In the present study, clinically isolated C22, bioluminescent PAO1, and CRPAO1, representing different resistance situations, were used to assess the antimicrobial efficacy of combinations.

As the basic antibiotic in combination, COL targets the nongrowing subpopulation in biofilms, which is unsusceptible to most other antibiotics. However, COL shows a poor effect on the actively growing subpopulation in biofilms (8, 14). Therefore, the combined use of antibiotics needs to meet the following criteria: the antibiotics can penetrate the biofilm and kill the cap subpopulation, which is active and COL-resistant. In our present study, we needed at least 8 mg/L COL to kill the living bacteria in the immature biofilm *in vitro*. For the infection of mature biofilm, we needed at least 16 mg/L COL, which was eight times of CLSI breakpoint. Such high concentrations cannot be achieved *in vivo*, primarily due to renal toxicity (15). *In vivo*, after 5 days of intraperitoneal injection of COL alone, the number of bacteria was only slightly decreased, and we could not completely eradicate it.

LEV can interfere with bacterial biofilm both during its synthesis and mature form (16). The possible mechanism includes electrostatic interference of the adhesion, activation or release of enzymes to disrupt the exopolysaccharide, and inhibition of the formation of new exopolysaccharides (17). Moreover, fluoroquinolones have produced bactericidal effects against stationary-phase pathogens, which is the main reason for recurrence (18). COL combined with ciprofloxacin has been shown to be efficient for the treatment of biofilms *in vitro*, which is attributed to the fact that the pattern of COL-mediated killing in biofilms is different from ciprofloxacin (8). In the present study for PAO1, we needed 4 mg/L and 8 mg/L of LEV alone (two and four times of CLSI breakpoint) to kill the bacteria in immature (1-day grown) and mature biofilms (3 and 7-day-grown) *in vitro*. Although 5 days of LEV monotherapy had some therapeutic effects on the PAO1 mouse model, the combination containing COL further shortened the clearance time to 4 days. However, for the C22 and CRPAO1, we needed at least 16 mg/L of LEV alone (eight times of CLSI breakpoint) to kill the bacteria in the mature biofilm. Even in combination with 2 mg/L COL, 8 mg/L LEV was still needed. Based on the *in vitro* results, it was not surprising that COL combined with LEV showed no advantage over LEV or COL alone when treating CRPAO1 biofilm *in vivo*. Generally, the effect of COL+LEV against CRPAO1 was weaker than PAO1. Since the MER-resistant strain is often resistant to LEV according to China Antimicrobial Surveillance (10), the application of COL+LEV in CR-PA is limited.

Although AMI has a high susceptible rate and good PK/PD (19), it lacks activity against cells in a slow-growth phenotype, and subinhibitory levels of aminoglycosides help induce the formation of biofilm (20). Therefore, the combination containing COL may help complement the shortcomings of both of them. In research on persister cells of Acinetobacter baumannii, COL combined with AMI shows a good synergy (21). COL combined with aminoglycoside-tobramycin shows a good effect in mice and patients with cystic fibrosis (14). Our results supported this idea. Single antibiotic therapy of AMI needed at least 64 mg/L (twice of CLSI breakpoint) to kill the living bacteria in the mature biofilm. *In vitro*, results showed that AMI at a concentration of 32 mg/L in combination with 2 or 4 mg/L COL could clear the living bacteria in biofilm in 24 h. COL+AMI also showed an excellent effect in an animal model, no matter the strains were PAO1 or MER-resistant PAO1.

Biofilms and device-related infections are commonly polymicrobial, and the selection of a broad-spectrum antimicrobial is preferred (22). Polymyxins have been assumed to play an essential role in the salvage therapy for otherwise untreatable Gram-negative infections, most notably multidrug-resistant (MDR) and XDR strains of PA, Acinetobacter baumannii, and Enterobacteriaceae (23). For LEV, it has broad-spectrum *in vitro* antimicrobial activity against Gram-negative and Gram-positive bacteria (24). Aminoglycoside antibiotics also have broad-spectrum bactericidal activity. However, AMI is known to develop resistance very slowly due to its complex structure (25). In the surveillance program, the susceptible rate of COL to MDR-PA can achieve 98.9% (*n* = 12,972), and the susceptible rate of LEV and AMI to MDR-PA is 62.3% and 64.1%, respectively (9, 10). Therefore, COL-based combinations containing LEV and AMI were promising in polymicrobial or MDR strain biofilm infection. However, there is one issue worth noting. Although 5 days of COL in combination with AMI showed slight nephrotoxicity in the mouse model according to pathological results in the preliminary experiments, the clinical use of two antibiotics with a high risk of nephrotoxicity requires careful evaluation.

### Conclusions.

COL-based combinations containing LEV or AMI had a synergistic activity against PA biofilm *in vitro* and *in vivo*, especially for those strains susceptible to both antibiotics.

## MATERIALS AND METHODS

### Strains and agents.

The bioluminescent strain PAO1 carrying *luxCDABE* gene operon was purchased from Caliper Life Sciences, USA. C1-C22 were clinically isolated from the Chinese PLA General Hospital and identified by the automated Vitek-2 system (bioMérieux, Marcy l’Etoile, France) with a rapid latex agglutination test. C22 was resistant to LEV, AMI, and sulfamethoxazole/trimethoprim. PA ATCC 27853 was used as the quality control strain.

AMK, MER, LEV (Shanghai Macklin Biochemical Co., Ltd., China), and COL sulfate (Sigma, China) were used in our present study. Mueller-Hinton Agar and adjusted Mueller-Hinton Broth (MHA/MHB, Becton, Dickinson and Company) were used to culture bacteria. Pentobarbital (Shanghai Rongchuang Biotechnology Company, China) and isoflurane (Shenzhen RWD Life Science Company, China) were used to anesthetize mice. LIVE/DEAD Bacterial Viability kit (Life Technologies Corporation, Oregon) was used to stain live and dead bacterial cells.

### Fractional inhibitory concentration index (FICI) assay.

A broth-microdilution method from the CLSI standards was employed to assess the MICs (26). Briefly, 2-fold serial dilutions of antibiotics were prepared in MHB at 100 μL per well in 96-well U-bottomed polystyrene microtiter plates (Corning/Costar, NY, USA). Each well was inoculated with 100 μL of the strain inoculum, yielding a final bacterial concentration of approximately 1 × 10^5^ CFU (CFU)/mL. The MICs were defined as the lowest concentration of the tested agent that resulted in the complete inhibition of visible growth in MHB. Minimum bactericidal concentrations (MBCs) were defined as the lowest antimicrobial concentrations that inhibited bacterial growth after the subculture of the suspensions on solid unselective media without any antimicrobial agent. Synergistic effects of antibiotics were assessed using the checkerboard broth microdilution method as previously described (27). The interactions between two tested antibiotics were evaluated by FICIs and calculated as follows: FICI = (MIC of drug A in combination/MIC of drug A alone) + (MIC of drug B in combination/MIC of drug B alone). The FICIs were interpreted as follows: FICI ≤ 0.5, synergy; 0.5 < FICI < 1.0, addictivity; 1 ≤ FICI ≤ 2.0, indifference; and FICI > 2.0, antagonism.

### Induction of MER resistance of PAO1.

The PAO1 was cultured in MHB containing 1/2 MIC MER. After 24 h, a susceptibility test was performed according to CLSI standards. The concentration of MER was repeatedly increased according to the MIC results in the broth until the PAO1 was resistant to MER. To test whether the characteristics of MER resistance and bioluminescence were stable, the MER-resistant PAO1 (CRPAO1) was subcultured five times, the MICs were assessed, and the IVIS Lumina III live animal biophotonic imaging system (PerkinElmer) was used to test bioluminescence.

### Minimum biofilm eradication concentrations (MBECs) *in vitro*.

The MBECs were determined as previously described (27). Briefly, biofilm was cultivated on disks in 24-well plates. The disks were cut from a medical drainage tube with a diameter of 0.5 cm. The plates were then incubated in MHB at 37°C for 1, 3, and 7 days, and MHB was renewed daily. Subsequently, the disks were washed thrice with MHB and put into new 24-well plates with different concentrations of antibiotics. After 24 h of treatment at 37°C, these disks were taken out and washed thrice with saline to remove planktonic bacteria. The adherent bacteria were collected from disks using an ultrasonic cleaning bath within 10 min. The bacterial solution was vigorously mixed, plated on agar plates as 10-fold serial dilutions, and cultured for 24 h. CFU was counted, and MBECs were calculated as the minimum concentrations of tested antibiotics to eradicate biofilm (CFU = 0).

### Biofilm imaging by confocal laser scanning microscopy (CLSM).

Specimens for CLSM were washed thrice in 0.9% physiological saline to remove the culture broth and planktonic bacteria. Biofilms were stained by LIVE/DEAD BacLight Bacterial Viability kit (catalog number L13152). The working solution was prepared according to the product information. The excitation/emission maxima for these dyes were about 480/500 nm for the SYTO 9 stain and 490/635 nm for propidium iodide. Fluorescence from the stained cell was viewed using a CLSM at a resolution of 1,024 × 1,024 pixels with a 10× lens. Simultaneous dual-channel imaging was used to display green and red fluorescences.

### Biofilm infection mouse model and treatment regimen.

PAO1 or CRPAO1 biofilm was grown on disks as described in the *in vitro* experiment. Next, ICR male mice weighing 25 to 30 g were anesthetized with an intraperitoneal injection of 1% pentobarbital (0.005 mL/g). Then the disks with biofilms were rinsed with sterile physiological saline solution and implanted subcutaneously at the dorsal midline as previously described (27). Mice were randomly divided into 12 groups with 10 mice in each group: control group (sterile physiological saline solution 10 mL/kg/24 h); COL group (COL 20 mg/kg/12 h); LEV group (LEV 32 mg/kg/24 h); LEV-2 group (LEV 64 mg/kg/24 h); AMI group (AMI 135 mg/kg/24 h); COL+LEV group (COL 20 mg/kg/12 h plus LEV 32 mg/kg/24 h); COL+LEV-2 group (COL 20 mg/kg/12 h plus LEV 64 mg/kg/24 h); and COL+AMI group (COL 20 mg/kg/12 h plus AMI 135 mg/kg/24 h). The dosage of COL was consistent with relevant literature reports (28). The dosages of AMI and LEV were determined according to the conversion of clinical human dosage to mice according to SmPC and FDA labels (24, 29). The Cmax values of LEV, LEV-2, AMI, and COL in our dosage were expected to be 2.8, 5.1, 60, and 16.8 mg/L, respectively (24, 29, 30). The antibiotics were injected intraperitoneally 24 h after implantation and continued for 5 days. All animal experiments and procedures were approved by the Animal Ethics Committee of Chinese PLA General Hospital (SQ2020095).

### Bio-photonic imaging of mouse biofilm model.

All mice were imaged immediately after implantation of disks with PA biofilm and every 24 h on IVIS Lumina Series III live animal biophotonic imaging system. Previous studies have demonstrated a good correlation between the luminescence of bacteria and bacterial counts (31, 32). Signals were collected from a defined region of interest (ROI) using the contour ROI tool, and total flux intensities (photons/s) were analyzed using Living Image Software 4.3.7 from 0 h postinfection to the end of therapy and disappearance of flux intensities. The sutures would be dehiscent in mice with uncured infection at about 10 days. Therefore, we set 10 days as the maximum observation time. When the total flux intensities (radiance) of ROI were less than 10^5^, the radiance of bacteria could not be distinguished from the background. After the last imaging, the implanted disks in all groups were taken out to examine the bacterial counts in biofilms. The method for counting biofilm bacteria was the same as the *in vitro* synergy test. A timeline representative of the experiments is shown in Fig. 1A.

### Renal histopathological examination.

The mice were injected intraperitoneally with antibiotics and continued for 5 days. In addition, one side of kidney tissue was formalin-fixed overnight, embedded in paraffin, sectioned into 5-μm slices, stained with hematoxylin-eosin (H&E), and examined under a Leica DME 100 light microscope.

### Statistical analysis.

All data were presented as mean ± standard error of the mean (SEM). Differences in log_10_ (CFU) counts among groups and log_10_ (p/s, photons/second) were assessed by one-way analysis of variance (ANOVA). Differences in the number of mice showing luminescence were assessed by Mantel-Cox. The changes of the photons from the 1st day to the 6th day were assessed by a Kruskal-Wallis test with Dunn's multiple comparison *post hoc* analysis. GraphPad Prism 8.0.2 was used for statistical analyses. *P* < 0.05 was considered statistically significant.
