# Supplementary material for: The Efficacy of Colistin Combined with Amikacin or Levofloxacin against Pseudomonas aeruginosa Biofilm Infection
Source: Microbiol Spectr. 2022 Sep 14;10(5):e01468-22. doi: 10.1128/spectrum.01468-22 (PMC9603716; doi:10.1128/spectrum.01468-22)

Fig. S1. *In vitro* synergistic activity of COL+AMI and COL+LEV combinations to 1-day-grown, 3-day-grown, and 7-day-grown PAO1 biofilms (A). Results represent means± SEM. CLSM images (B) of PA biofilm treated with control (MHB), COL (2 mg/L), AMI (8 mg/L for 1 day, 32 mg/L for 3 days and 7 days), LEV (2 mg/L for 1 day, 4 mg/L for 3 days and 7 days), COL+AMI (4 mg/L+8 mg/L for 1 day, 2 mg/L+32 mg/L for 3 days and 7 days), and COL+LEV (2 mg/L+2 mg/L for 1 day, 2 mg/L+4 mg/L for 3 days and 7 days). Clinical breakpoints were marked with lower horizontal lines.

A

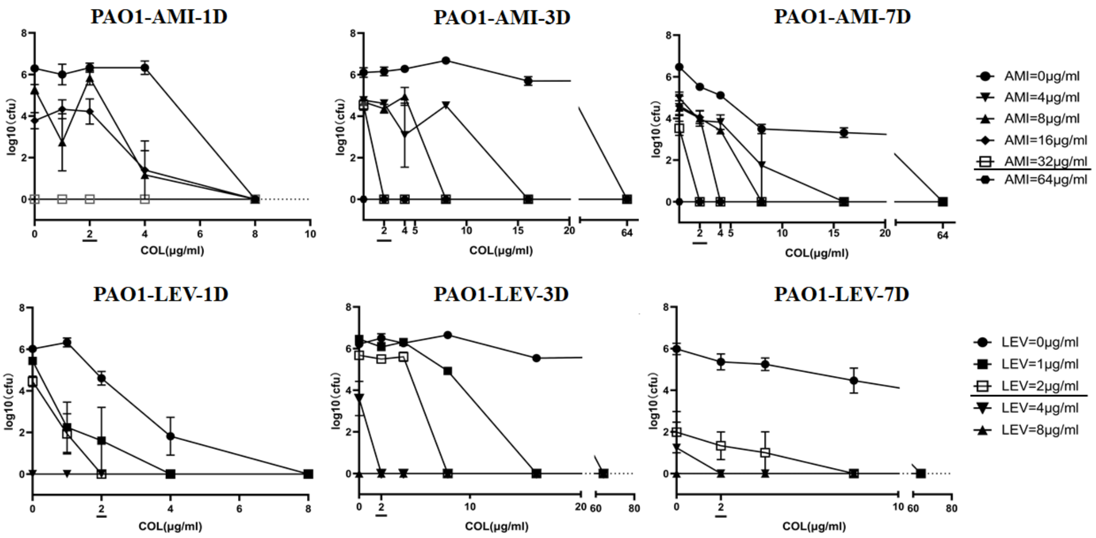

B

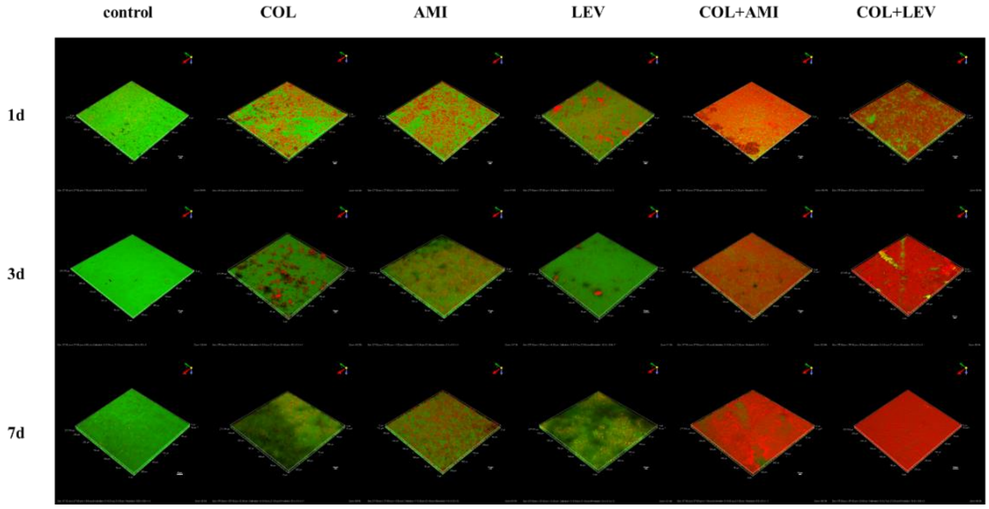

Fig. S2. *In vitro* synergistic activity of antimicrobial combinations to CRPAO1 1-day-grown, 3-day-grown, and 7-day-grown biofilms (A). Results represent means± SEM. CLSM images (B) of biofilm treated with control (MHB), COL (1 mg/L for 1 day, 2 mg/L for 3 days, 4 mg/L for 7 days), AMI (32 mg/L), LEV (4 mg/L for 1 day, 8 mg/L for 3 days and 7 days), COL+AMI (1 mg/L+32 mg/L for 1 day, 2 mg/L+32 mg/L for 3 days, 4 mg/L+32 mg/L for 7 days), and COL+LEV (1 mg/L+4 mg/L for 1 day, 2 mg/L+8 mg/L for 3 days and 7 days). Clinical breakpoints were marked with lower horizontal lines.

A

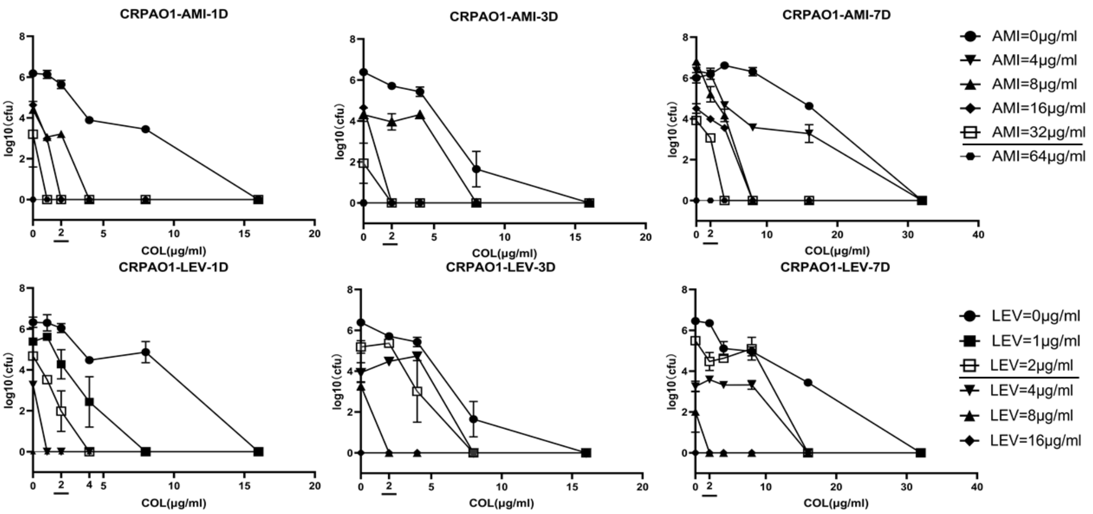

B

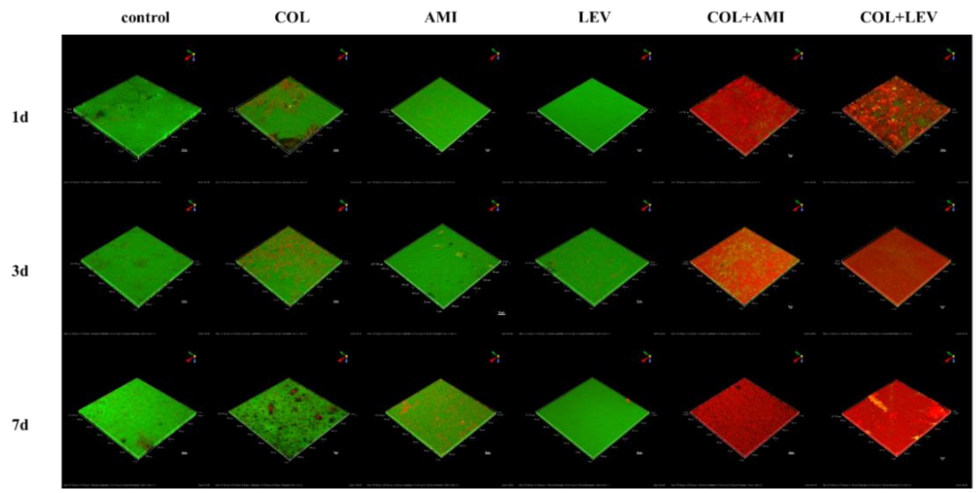

Fig. S3. *In vitro* synergistic activity of COL+LEV combination to C22 1-day-grown, 3-day-grown, and 7-day-grown biofilms (A). Results represent means± SEM. CLSM images (B) of PA biofilm treated with control (MHB), COL (2 mg/L), LEV (8 mg/L for 1 day, 16 mg/L for 3 days, 32 mg/L for 7 days), and COL+LEV (2 mg/L+8 mg/L for 1 day, 2 mg/L+16 mg/L for 3 days, 2 mg/L+32 mg/L for 7 days). Clinical breakpoints were marked with lower horizontal lines.

A

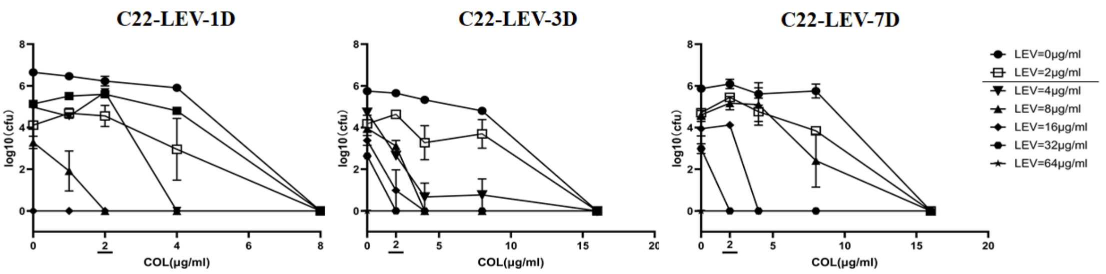

B

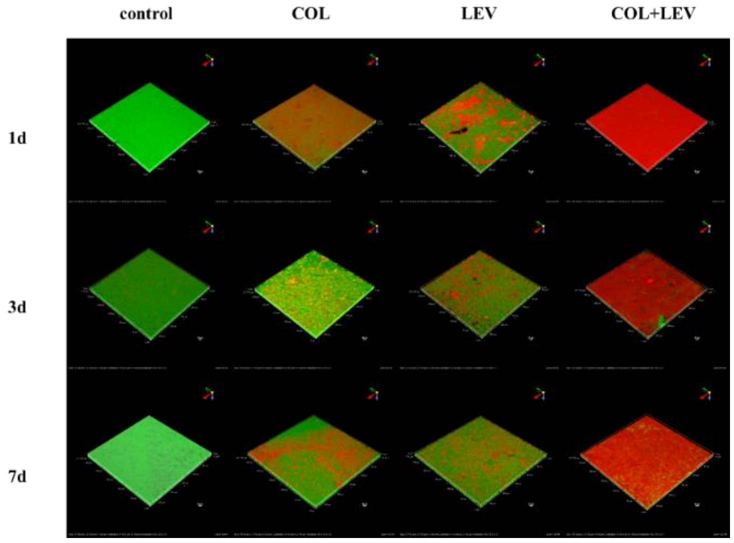

Fig. S4. CFU counts on the 10th day. Biofilms were harvested from the animal model, and bacteria were collected. Missing data exploration: wounds of several mice were dehiscent, and implants were partially exposed. Results represent as means± SEM.

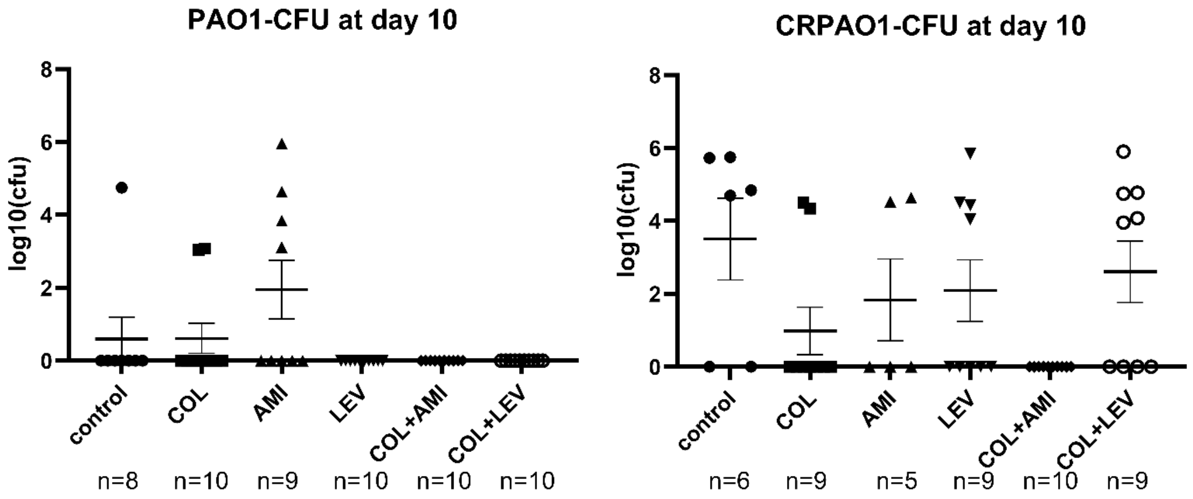

Fig. S5. Histological images (H&E, ×200) of kidney tissues in mice. Vacuolar degeneration of renal tubular epithelial cells (yellow arrow). Renal tubule dilatation (black arrow). The mice were treated with COL (20 mg/kg, bid), AMI (135 mg/kg, qd) and COL+AMI (20 mg/kg, bid+135 mg/kg, qd).

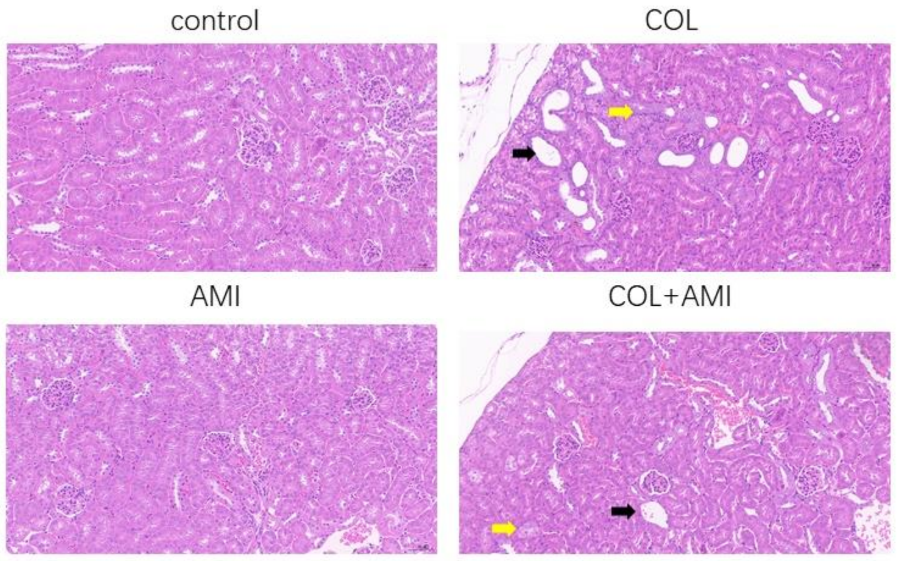

Supplement: Supplemental file 1 — Supplemental material. Download spectrum.01468-22-s0001.pdf, PDF file, 0.6 MB [file spectrum.01468-22-s0001.pdf]
